# Rashba Splitting and Electronic Valley Characteristics of Janus Sb and Bi Topological Monolayers

**DOI:** 10.3390/ijms23147629

**Published:** 2022-07-10

**Authors:** Qi Gong, Guiling Zhang

**Affiliations:** School of Material Science and Chemical Engineering, Harbin University of Science and Technology, Harbin 150040, China; gongqi.hrbust@gmail.com

**Keywords:** two-dimensional Janus structure, topological insulator, quantum spin Hall effect, valley electronics

## Abstract

Janus Sb and Bi monolayers as a new class of 2D topological insulator materials, which could be fulfilled by asymmetrical functionalizations with methyl or hydroxyl, are demonstrated by first-principles spin–orbit coupling (SOC) electronic structure calculations to conflate nontrivial topology, Rashba splitting and valley-contrast circular dichroism. Cohesive energies and phonon frequency dispersion spectra indicate that all Janus Sb and Bi monolayers possess a structural stability in energetic statics but represent virtual acoustic phonon vibrations of the hydrogen atoms passivating on monolayer surfaces. Band structures of Janus Sb and Bi monolayers and their nanoribbons demonstrate they are nontrivial topological insulators. Rashba spin splitting at G point in Brillouin zone of Janus Bi monolayers arises from the strong SOC *p*_x_ and *p*_y_ orbitals of Bi bonding atoms together with the internal out-of-plane electric field caused by asymmetrical functionalization. Janus Sb and Bi monolayers render direct and indirect giant bandgaps, respectively, which are derived from the strong SOC *p*_x_ and *p*_y_ orbitals at band-valley Brillouin points K and K′ where valley-selective circular dichroism of spin valley Hall insulators is also exhibited.

## 1. Introduction

Two-dimensional (2D) topology insulators and nanomaterials have gained more attention since graphene was exfoliated successfully, and the recently focused Rashba effects and valleytronics promote emergence of various practical spintronic devices, especially for operating at room temperature [1,2]. Janus polar-structures have attracted intensive attention due to their specific intrinsic properties and potential applications of exploiting Janus anisotropy in spintronic devices, catalysts, drug delivery, biosensors and antibacterials [3,4,5]. Two-dimensional Janus discs have been demonstrated both in theory and practice to provide new schemes of modifying electronic structure and surface functionality of graphene-like 2D materials [6,7,8]. Spin–orbit coupling (SOC) plays an important role together with internal built-in electric fields in Janus quantum systems with a 2D honeycomb atomic structure to cause various physical effects, such as Rashba spin-splitting and electronic valley freedom from Dirac-point electronic states at Brillouin zone boundary [9,10].

Surface functionalizations of physical or chemical decorations on 2D graphene-like materials provides a new routine of manipulating electronic properties by band structure engineering. Physical adsorption of gas or small organic molecules provides a rather feasible method to achieve preferable electronic properties, which will generally cause substantial reconstruction in local atomic structures to engender great changes in electronic structures [11,12,13]. However, covalent functionalization is a better way of acquiring higher stability and homogeneity. Previous researches on the covalent functionalizations of elemental 2D topological materials incorporate hydrogenation and halogenation, oxidation, organic group decoration and cycloaddition reaction [14,15,16,17,18,19]. The plasma method of halogenation leads to a serious increase in lattice defects and thus even ruins topological properties, and the hydrogenated surface is easy to be oxidized at room temperature due to the poor chemical stability [20,21].

Extensive effort has been devoted to the search for new 2D topological insulators (TIs) with a large bulk band-gap. Some layered materials such as silicene, germanene and stanene have been proposed, and the bulk band-gap of 2D TIs has been promoted remarkably to 0.3 eV in chemically modified tin films [22,23,24,25]. Recently, ultrathin Bi films have drawn much attention as promising candidates for quantum spin Hall effect (QSH) insulators, and the 2D topological properties of ultrathin Bi(111) films have been reported [26]. To the best of our knowledge, neither 2D nor 3D TIs have achieved a bulk band-gap exceeding 0.7 eV [27]. Because Bi and Sb are well known for their strong SOC that can generate and stabilize topologically nontrivial electronic-states, it is worthwhile to explore large band-gap QSH insulators from Bi- or Sb-based 2D materials, such as Sb or Bi monolayers symmetrically functionalized by methyl, amino and hydroxyl groups [28]. Especially when the inversion symmetry of honeycomb lattice is broken, Bi and Sb monolayers become a quantum valley Hall insulator, whilst Rashba spin splitting and chiral optical selectivity of electronic valleys are expected to be feasibly achieved. To date, no research has been reported focusing on the asymmetrical functionalization of Sb or Bi monolayers to realize these versatile exotic phenomena from nontrivial topology in inversion asymmetry and fabricate new quantum devices operating at room temperature. Such novel structures will promote graphene-like elemental 2D topological materials to a controllable and practical level of fulfilling advanced applications in innovative 2D spintronic devices.

In the present study, we intensively investigate the asymmetrical (Janus) functionalizations of Sb and Bi monolayers by chemically attaching hydrogen atoms on one side and chemical groups (methyl or hydroxyl) on the other side according to the so-called “chair” prototypical geometry [29]. First-principles calculations of structure optimization, phonon dispersion and electronic structures are carried out to theoretically verify that Sb and Bi monolayers asymmetrically functionalized by methyl or hydroxyl are stable giant band-gap QSH insulators with a giant bulk band-gap. It is suggested that their giant bulk band-gaps originate from strong SOC near Fermi level that is dominantly contributed by Sb or Bi *p*_x_ and *p*_y_ orbitals in Janus monolayers, which makes them promising for topological insulator applications at ambient temperature. Meanwhile, when the inversion symmetry of Bi or Sb monolayers in honeycomb lattices is broken by asymmetrical functionalization of hydrogen and methyl or hydroxyl respectively on two surface sides, we obtain Janus 2D structures with evident Rashba splitting and chiral optical selectivity.

## 2. Results and Discussion

### 2.1. Atomic Geometry and Stability

**Figure 1:**
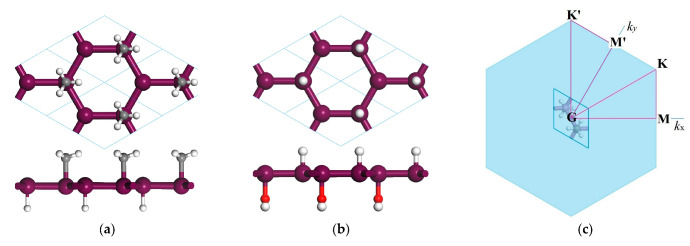
(**a**,**b**) Top-view (top) and side-view (bottom) schematics of Janus Sb or Bi monolayers with asymmetrical decorations of H and CH_3_ or OH on two surface sides, respectively, where the violet, gray, red and white balls symbolize the bonding atoms of Sb or Bi, C, O and H, respectively; (**c**) high-symmetry points (M, K, M′, K′) specifying the dispersion paths of phonon frequency or electron energy in Brillouin zone.

Janus Sb or Bi monolayers (HSb_2_XH_n_ and HBi_2_XH_n_, XH_n_ = CH_3_, OH) are obtained by asymmetrical functionalizations (decorations) where the radical groups of methyl (CH_3_) and hydroxyl (OH) are bonded to Sb or Bi atoms on one surface side of Sb or Bi monolayer with the other surface side being saturated by hydrogen (H), as schematically shown in Figure 1, which possess space group of P3M1 in a threefold rotational symmetry of 2D honeycomb lattices. Compared with Sb or Bi and their hydrogen-saturated monolayers, the inversion symmetry has been broken for Janus Sb or Bi monolayers due to asymmetrical decoration. Lattice constants, bond lengths of Sb or Bi with center atoms of CH_3_ or OH, center buckle thickness, entire layer thickness, cohesive energy, Mulliken population charge of Janus Sb or Bi monolayers are listed in Table 1. We can see the appreciable buckle configurations of central Sb or Bi atomic layers for SbH or BiH monolayer and the quasi-planar geometry of Janus structures (for comparison). It has been demonstrated by preliminary calculations that the cohesive energy of the SbH or BiH monolayer (without chemical group decoration and no Janus) is higher than that of their purely elemental counterparts. Even though the bonding strength of Sb or Bi with C or O atoms is substantially higher than that with H atoms, the remarkably larger buckling of SbH and BiH monolayers leads to a comparable and even higher cohesive energy as that of Janus Sb and Bi monolayers, as shown in Table 1. The bonding strength, which is characterized by bond length and cohesive energy, increases with the increase in electronegativities of C and O elements. In particular, the cohesive energy approaches the highest and lowest values of 9.59 and 7.53 eV/unitcell for HSb_2_OH and HBi_2_CH_3_, respectively. It is an energetic indication that Janus Sb or Bi monolayers can be realized by strongly bonding CH_3_ or OH with Sb or Bi atomic layer into perfect crystalline structures. Thus, it is preferential to synthesize these asymmetrically functionalized monolayers with a high static stability in structure.

**Figure 2:**
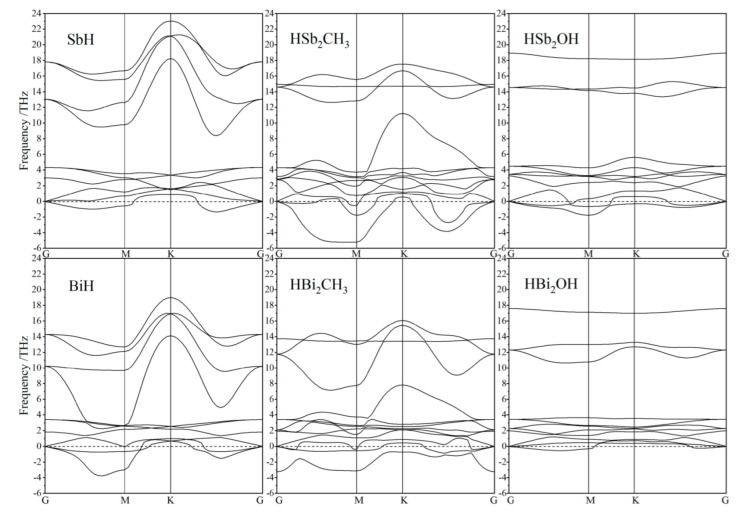
Phonon dispersion spectra of Janus Sb and Bi monolayers in comparison to SbH and BiH monolayers.

Kinetic stability under atomic oscillations is evaluated with frequency dispersion spectra of phonon modes, as shown in Figure 2. Preliminary phonon calculations by the linear response method [30] indicate that LO–TO frequencies at Brillouin zone center are degenerated for all these Janus and SbH or BiH monolayers, verifying that LO–TO splitting can be neglected. Phonon dispersion spectra of Janus Sb and Bi monolayers represent 12 branches comprised of three acoustic and nine optical branches. Acoustic phonon modes are classified into longitudinal acoustic waves, in-plane and out-of-plane transverse acoustic waves, while optical phonons contain an isolated out-of-plane transverse optical wave and a pair of G-point degenerated longitudinal and in-plane transverse optical waves. Both Janus Sb or Bi monolayers and SbH or BiH monolayers represent partially negative acoustic phonon frequencies in dispersion along G–M while approaching positive values near K point, which is attributed to virtual vibrations of H atoms passivating on Sb or Bi atomic layers. This result suggests that it is preferable to use other elements with a heavier mass and higher bonding strength rather than H atoms to saturate *p*_z_ orbitals of Sb or Bi monolayers for complete kinetic stability.

### 2.2. Internal Gradient of Electrostatic Potential

**Figure 3:**
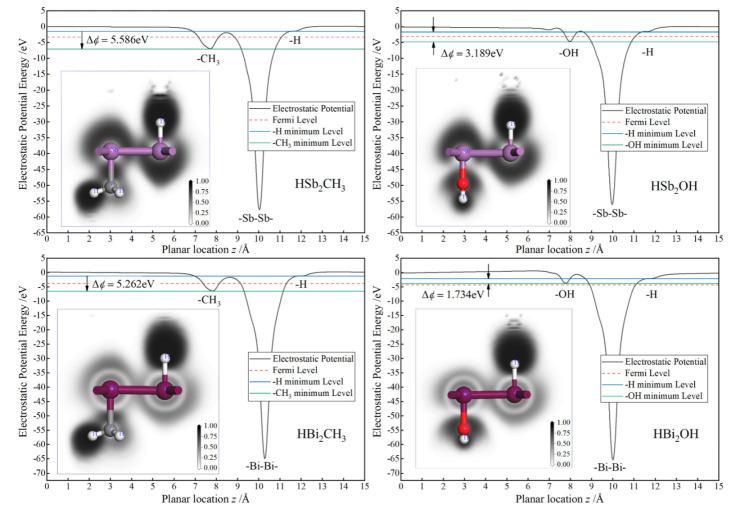
Internal potential gradient and bonding characteristics of the Janus Sb and Bi monolayers: mean electrostatic potential as a function of the planar position, with the inserted panels showing the electron localization function contoured on the normal plane crossing both Sb or Bi and C or O atomic centers.

Mulliken population charges on the C and O bonding atoms of −0.5 and −0.7 e (or −0.9 and −1.3 e), respectively, due to their higher electronegativity than that of Sb (or Bi) are in contrast to −0.1 e (or 0.7 e) on hydrogen atoms directly bonding with Sb or Bi atoms, as listed in Table 1, implying substantial charge transfer and electronic cloud shifting out of central layer plane in Janus Sb and Bi monolayers. Accordingly, significant internal electric fields of Janus monolayers are created by the potential differences of ∆*ϕ* = 5.586, 3.179, 5.262 and 1.734 eV for HSb_2_CH_3_, HSb_2_OH, HBi_2_CH_3_ and HBi_2_OH, as shown in Figure 3. Therefore, it is suggested that inversion symmetry of Sb and Bi monolayers has been broken in these Janus monolayers due to the asymmetric decoration of CH_3_ or OH. For the Janus monolayers based on a heavy main group element, the electronegativity of center atoms in asymmetrically decorating chemical groups is a judgement giving rise to an intrinsic electric field.

Bonding characteristics of Sb or Bi with C or O atoms are evaluated by electron localization function (ELF), as depicted with the white–black contoured maps on the normal plane through atomic centers in the inserted panels of Figure 3. For all these Janus Sb and Bi monolayers, the highest ELF appears around the bonding middle between C atom center of CH_3_ and Sb or Bi atoms as a manifestation of covalent bonding in consistence with the minimal electron transfer according to Mulliken population charges. By contrast, for H or O atoms bonding with Sb or Bi atoms, the high ELF region resides around H or O center and promptly decreases at the middle of bonds, which implies ionic character, accounting for an internal electric field internal Janus Sb or Bi monolayers. ELF distributions reveal the chemical bonding attributes of the internal electric fields caused by asymmetrical functionalization with CH_3_ or OH.

### 2.3. Band Structure

**Figure 4:**
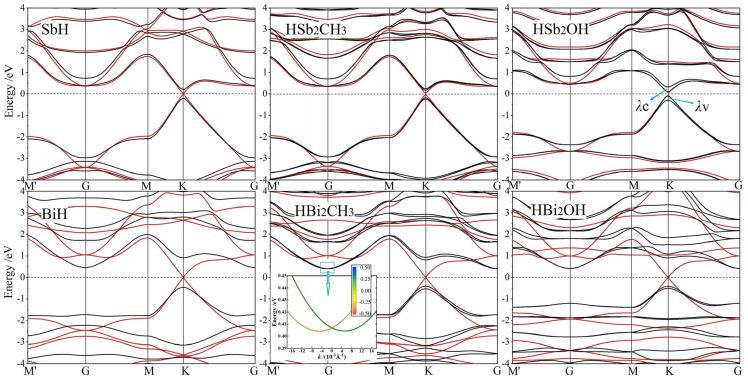
Band structures without SOC (red curves) and with SOC (black curves) of Janus Sb (top panels) and Bi (bottom panels) monolayers, and SbH and BiH monolayers with inversion symmetry are presented for comparison, in which Fermi level is referenced as energy zero (horizontal dash line).

All the band structures of Janus Sb and Bi monolayers from our calculations are merged in Figure 4, where the SOC effect could be distinguished by red and black dispersion curves. According to partial and atomic projections, it is determined that band-edge electronic-states near Fermi level are primarily composed of *p*_x_ and *p*_y_ orbitals from Sb or Bi bonding atoms [31]. Even without SOC, the highest valence band and the lowest conduction band crossing linearly at K and K′ points imply the Dirac-cone-like characteristics of these 2D honeycomb systems. When SOC is included, the degeneracy at Dirac-cone point splits off with the highest valence band and the lowest conduction band shifted towards high and low energy from Fermi level, respectively, thereby opening a notable band-gap by SOC for both the symmetrically hydrogenated and Janus monolayers. By contrast, at G point, the valence and conduction bands are upshifted and downshifted respectively, which accounts for a global indirect band-gap in the functionalized Bi monolayers with a remarkably larger K-point splitting caused by a much stronger SOC of Bi systems than that of Sb systems. Global band-gaps of Janus Sb and Bi monolayers range in 0.2~0.4 eV and 0.8~0.9 eV, respectively, as listed in Table 2.

It has been reported by ab-initio calculations that Rashba spin-splitting occurs in all the Janus Mo and W dichalcogenide monolayers due to the simultaneous existences of strong SOC and internal electric field. Consistently, Rashba spin-splitting arises in HBi_2_CH_3_ monolayers with a built-in potential gradient as shown in Figure 3, leading to a perceptible Rashba parameter *R*_G_ of 0.224 eV·Å due to the strong SOC from *p*_x_ and *p*_y_ orbitals of Bi bonding atoms, as manifested by comparing non-SOC and SOC band structures in Figure 4. It is suggested by the lower built-in electric field in Rashba splitting HBiCH_3_ than in HSb_2_CH_3_ with a lower SOC that strong SOC dominates the generation of Rashba splitting in Janus Sb and Bi monolayers. Since SOC and angular momentum cannot be simultaneous represented in quantum mechanics, the spin-up and spin-down states cannot be completely determined in SOC band structures, as shown by the color-mapped spin components near Fermi level for Rashba HBiCH_3_ monolayer in the inserted panel of Figure 4. Meanwhile, SOC also causes remarkable band-edge splittings at K point for Janus Sb and Bi monolayers, as indicated by *λ*v and *λ*c for the upmost valence band and the downmost conduction band, respectively, with the values listed in Table 2. For HBi_2_CH_3_ and HBi_2_OH monolayers, the valley shape of the upmost valence band is preferred over the flat shape of the downmost conduction band at K point for valleytronics applications.

Topological properties of SbH and BiH monolayers are characterized by the topological Z_2_ invariant evaluated from first-principles electronic Bloch wave-functions, where Z_2_ = 1 and Z_2_ = 0 represent the nontrivial and trivial topology, respectively. Since the atomic structures of SbH and BiH monolayers have space inversion symmetry, the Z_2_ topological invariant is directly calculated by Bloch wave-function parities of valence electrons at time-reversal-invariant points (TRIPs) in Brillouin zone. Electronic structures of SbH and BiH monolayers have four TRIPs, one at G point and three at M point, so the topological index *P* can be calculated as follows:

(1) $$P(K_{i})=\prod_{m=1}^{N}\delta_{2m}^{i}\begin{array}{cc} , & {\left(-1\right)}^{\nu }=\prod_{i=1}^{4}P(K_{i})= \end{array}P(G)P{\left(M\right)}^{3}$$

where *P* denotes the parity product of Bloch wave-functions on TRIPs, *δ* = ±1 indicates parity, and *N* is the number of valence bands. The 12 valence electrons in primitive cells of SbH or BiH monolayer constitute six spin-degenerate electronic-states at a TRIP, and the parities of spin-degenerate electron eigenfunctions of SbH and BiH monolayers on all TRIPs are identical. As shown in Table 3, the values of *P* at G and M points are –1 and +1 respectively, which leads to the nontrivial Z_2_ = 1, demonstrating that SbH and BiH monolayers are nontrivial topological insulators.

**Figure 5:**
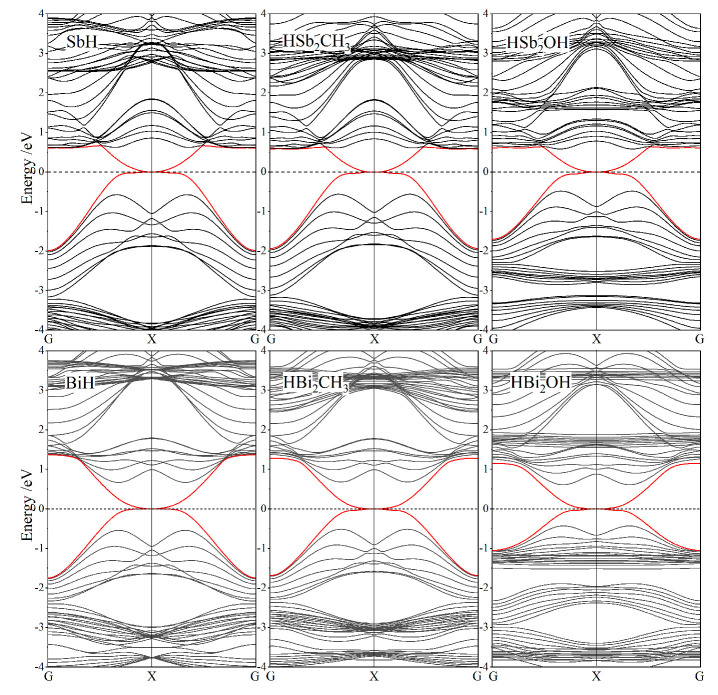
SOC-included band structures of zigzag nanoribbons of Janus Sb and Bi monolayers (functionalized asymmetrically by H atom and CH_3_ or OH), and SbH and BiH nanoribbons are presented for comparison, with red color indicating edge states and Fermi level being referenced as energy zero.

In contrast, it is invalid of evaluating Z_2_ invariant by the parity of valence electronic-states to determine whether Janus Sb and Bi monolayers are topologically trivial or nontrivial, because they are lacking of space inversion symmetry due to the asymmetrical functionalization of chemical groups. Nevertheless, nontrivial topology of Janus Sb and Bi monolayers could be represented in the odd number of conductive channels from edge electronic-states without band-gap, where Dirac-cone point appears to be crossed by the lowest conduction band and the highest valence band of nanoribbon at Fermi level. To investigate this important topological property, the zigzag nanoribbons of Janus Sb and Bi monolayers are constructed, in which atomic structures on both edges are mirror-symmetrical to each other, and the unsaturated edge bonds are passivated by hydrogen atoms. Nanoribbon width is specified about 35 Å to avoid overlapping between two edge states of nanoribbon. After geometrical optimization, the band structures of Janus Sb and Bi monolayer nanoribbons are calculated, with the results shown in Figure 5. The energy dispersion of edge electronic-states of these nanoribbons intersects into a Dirac-cone point of Fermi level at boundary X point of one-dimensional Brillouin zone. The odd number of edge-state conductance channels is derived from nontrivial topology that will also present edge-state Dirac-cones in band structures of armchair nanoribbons. Therefore, this Dirac-cone signature of edge states proves these Janus Sb and Bi monolayers are (nontrivial) topological insulators to realize QSH effect at ambient temperature supported by their giant band-gaps.

### 2.4. Valley-Contrast Circular Dichroism

**Figure 6:**
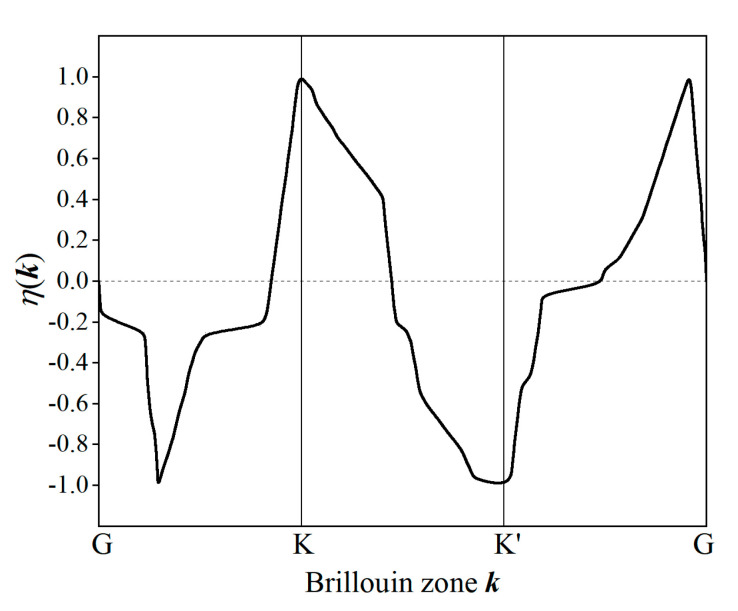
Valley polarization of HBi_2_OH monolayer along high-symmetry path through valley K and K′ points.

There is a valley degree of freedom in Janus Sb and Bi monolayers, and the strong SOC opens a large electronic energy gap at the corners of Brillouin zone. Inversion symmetry is broken in Janus Sb and Bi monolayers due to the asymmetrical functionalizations with CH_3_ or OH, leading to valley-contrast Berry curvature and spin that yield valley spin Hall effect and valley-contrast circular dichroism. With a giant bulk band-gap and nontrivial topology, these Janus monolayers can afford to be a quantum spin Hall insulator and a quantum valley Hall insulator at ambient temperature. Valley-contrast Berry curvature and spin can be described by valley polarization *η*(***k***) in Brillouin zone as follows:

(2) $$\eta (k)=\frac{{\left|P_{+}(k)\right|}^{2}-{\left|P_{-}(k)\right|}^{2}}{{\left|P_{+}(k)\right|}^{2}+{\left|P_{-}(k)\right|}^{2}},\begin{array}{cc} & P_{\pm } \end{array}=P_{x}\pm iP_{y}$$

where *P*_α_ (α = *x*, *y*) denotes momentum matrix element between the lowest conduction band and the highest valence band. Prototypically, the calculated *η*(***k***) of HBi_2_OH monolayer along the high-symmetry path through valley K and K′ points in Brillouin zone is shown in Figure 6, illustrating the contrast of optical selection rule between two valley counterparts. The left-hand and right-hand circular polarized photons are almost purely absorbed at K and K′ valleys, respectively. Therefore, circular polarized photons can distinguish electronic excitation and imbalance electron occupation between the two inversely distributed nonequivalent electronic-valleys in Janus Sb and Bi monolayers without external electric field.

According to a low-power model, the valley magnetic moments in Janus Sb and Bi monolayers are related to both valley pseudo-spin and real spin which are substantially coupled due to giant band-gaps induced by strong SOC. Valley pseudo-spin can be rendered by strong SOC under the internal built-in electric field in Janus Sb and Bi monolayers, which is different from inversion symmetry systems that need an external electric field. Notably, valleytronics can hardly be achieved due to minimal SOC in graphene or silicene, whilst requires a high external electric field to break inversion symmetry in SbH and BiH monolayers.

## 3. Theoretical Methodology

Employing the fully relativistic pseudopotential planewave first-principles method based on spin density functional theory, the atomic structure, phonon dispersion, SOC electronic structures of Janus Sb and Bi monolayers are calculated as implemented by CASTEP module of Materials Studio 2020 package (Accelrys Inc., Materials Studio version 2020.08, San Diego, CA, USA) [32]. Gradient-corrected exchange–correlation functional Wu and Cohen (WC) is adopted to perform the electronic state and energy calculations [33]. WC functional has been shown to achieve a significant improvement over LDA, PBE and TPSS functionals for predicting atomic structures of solids including ionic compounds and describing covalent bonds of molecules, especially for group I, group IV and group III–V semiconductors [34]. SOC is incorporated into the calculations of energy, atomic force and geometry, electronic structure and optoelectronic matrix elements. Band structures without SOC are also calculated for comparison to reveal the SOC-induced source of Rashba splitting, valley freedom and giant band-gaps.

Interactions between atomic cores and valence electrons are presented by norm-conserving pseudopotential, and the relativistic effect is treated with Koelling–Harmon schemes [35]. Electron wave functions are expanded by plane-wave basis-set under 720 eV energy cutoff, whilst finite basis-set correction is used in energy and stress calculations by estimating energy derivatives from three-points numerical differentiation [36]. Pulay density mixing scheme at 0.5 charge magnitude is utilized to carry out self-consistent field (SCF) iterations with a convergence tolerance of 5 × 10^−7^ eV/atom and on an FFT grid of 48 × 48 × 200 for electron density calculations [37,38]. Monkhorst–Pack 3 × 3 × 1 grid of *k*-sampling is adopted for integration in Brillouin zone [39].

Two-dimensional atomic structures are modeled in virtual three-dimensional space by setting a sufficient vacuum layer (15 Å) between the periodic images of monolayers. Geometry optimization is fulfilled by minimizing energy functional with BFGS algorithm in delocalized internal coordinates instead of Cartesian coordinates, so as to reach the energy, atomic force and stress convergences of 5.0 × 10^−6^ eV/atom, 0.01 eV/Å and 0.02 GPa, respectively [40]. Phonon structures are calculated by finite-displacement method using several small supercells without SOC-induced bonding force and ignoring frequency splitting between the longitude and transverse optical vibration modes (LO–TO splitting) at G point, in which the convergence tolerance of mechanical constant is specified as 1.0 × 10^−5^ eV/Å^2^.

## 4. Conclusions

Spin–orbit coupling electronic structures of Janus (asymmetrically functionalized by CH_3_ or OH) Sb and Bi monolayers (HSb_2_XH_n_ and HBi_2_XH_n_) with nontrivial topology, Rashba splitting and valley-contrast circular dichroism are first proposed and demonstrated in the present study. Cohesive energies and phonon dispersion indicate that all these Janus systems have innate static stability, whilst undergoing kinetic instability of H atoms capping another surface of Sb or Bi atomic layer. Under SOC effects, Janus Sb and Bi monolayers show direct and indirect band-gap characteristics of band structures, respectively. It is identified by means of first-principles calculations that HSb_2_XH_n_ and HBi_2_XH_n_ are a new family of giant band-gap 2D TIs with the largest bulk band-gap of ~0.8 eV, by far exceeding the current band-gaps of 2D TI materials realized in experiments. These giant band-gaps are entirely attributed to strong SOC of Sb or Bi *p*_x_ and *p*_y_ atomic orbitals around K and K′ valleys in Brillouin zone of honeycomb lattice, which suffice for practical applications at ambient temperature. Rashba splitting originates dominantly from strong SOC together with built-in out-of-plane electric field, as manifested by the unique Rashba HBi_2_CH_3_ monolayer possessing strong SOC but with a minimal out-of-plane potential gradient. Janus Sb and Bi monolayers are identified to be a spin valley Hall insulator by valley-selective circular dichroism. Due to strong SOC, the valley magnetic moments could be modified by intrinsic out-of-plane electric field to produce a substantial correlation between real and valley-pseudo spins. These results represent a significant advance in 2D TIs to stimulate further work of developing these new 2D TIs for Rashba spintronic devices and quantum valley Hall insulators.

## Figures and Tables

**Table 1:** Lattice constants *a*, bond lengths of Sb or Bi with center atoms of CH_3_ or OH (*d*_MX_), center buckle thickness (*h*_M_), entire layer thickness (*h*), cohesive energy (*E*_coh_), Mulliken population charge (*q*_M_) where M_1_ and M_2_ indicate two atoms of Sb or Bi and X denotes the functional-group center atom (C or O) or the H atom (for SbH and BiH only) of Janus Sb and Bi monolayers together with the SbH and BiH monolayers for comparison.

Monolayers	*a*/Å	*d*_MX_/Å	*h*_M_/Å	*h*/Å	*E*_coh_/eV·unitcell^−1^	*q*_M_/e		
						M_1_	M_2_	X
SbH	5.218	1.719	0.092	3.346	9.409	0.13	0.13	−0.13
HSb_2_CH_3_	5.207	2.173	0.003	4.252	9.245	0.29	0.09	−0.53
HSb_2_OH	5.219	1.912	0.003	4.596	9.591	0.71	−0.13	−0.71
BiH	5.467	1.851	0.175	3.526	7.771	0.73	0.73	−0.73
HBi_2_CH_3_	5.478	2.333	0.046	4.475	7.534	1.08	0.48	−0.93
HBi_2_OH	5.494	2.120	0.034	4.656	7.619	1.18	0.46	−1.32

**Table 2:** Rashba spin-splitting *R*_G_ of the highest valence band at G point, SOC-induced splitting *λ*v and *λ*c at K point for valence and conduction band-edges respectively, SOC-induced Dirac-cone splitting gaps at K point *E*_D_(K), global band-gaps *E*_g_(global) of Janus Sb and Bi monolayers compared with SbH and BiH monolayers.

Monolayers	*R*_G_/eV·Å	*λ*v/meV	*λ*c/meV	*E*_D_(K)/eV	*E*_g_(global)/eV
SbH	0	0	0	0.426	0.426
HSb_2_CH_3_	0	0.060	0.065	0.351	0.351
HSb_2_OH	0	0.194	0.229	0.194	0.194
BiH	0	0	0	1.372	0.918
HBi_2_CH_3_	0.224	0.124	0.123	1.219	0.809
HBi_2_OH	0	0.093	0.177	1.323	0.824

**Table 3:** Parities *δ* and their product *P* of spin-degenerate states at TRIPs for SbH and BiH monolayers.

TRIP	Parity *δ*						*P*
G	+1	+1	−1	+1	−1	+1	−1
3 M	+1	−1	+1	−1	+1	−1	+1

## Data Availability

Theoretical methods and results are available from all authors.
